# Levothyroxine before conception in women with thyroid antibodies: a step forward in the management of thyroid disease in pregnancy

**DOI:** 10.1186/s13044-019-0066-0

**Published:** 2019-06-20

**Authors:** Roberto Negro

**Affiliations:** Division of Endocrinology, “V. Fazzi” Hospital, Piazza F. Muratore, 73100 Lecce, Italy

**Keywords:** Thyroid, Autoimmunity, Pregnancy, Miscarriage, Preterm delivery, Hypothyroidism, Thyroperoxidase antibodies

## Abstract

Studies suggesting an association between thyroid autoimmunity and pregnancy-related adverse outcomes, particularly miscarriage and preterm delivery, date to nineties. The postulated causes for these associations were attributed to a direct or indirect effect of autoimmunity and/or to a mild thyroid impairment.

Since then, small trials and several meta-analyses confirmed a detrimental effect of thyroid autoimmunity and suggested that patients with thyroid autoimmunity who wish to conceive or are pregnant might benefit from levothyroxine treatment to decrease the rate of miscarriage and preterm delivery.

A recently published large trial investigated the hypothesis that the administration of levothyroxine in euthyroid antibody-positive women seeking pregnancy might increase the live birth rate.

Women who were trying to conceive and had a history of miscarriage or infertility were tested for TSH and thyroperoxidase antibodies. Euthyroid antibody positive women were randomized to receive 50 μg/day of levothyroxine or placebo and were tested for thyroid function throughout pregnancy. In patients with thyroid function test results outside of assay-specific reference limits, the trial agent was discontinued. 56.6% in the LT4 group and 58.3% in the placebo group became pregnant and the live birth rates were similar in the two groups (37.4% vs 37.9%, respectively). There was also no difference in pre-term delivery rate and other maternal and neonatal outcomes between the two groups.

The present commentary discusses the main findings of the trial and implications for clinical practice.

## Background

The first study showing an association between thyroid antibodies and increased rate of miscarriage dates back to the nineties when Stagnaro-Green, evaluating incidence and causes of post-partum thyroiditis, accidentally found that women positive for thyroperoxidase antibodies (TPOAb), miscarried twice as frequently compared to TPOAb negative women [1]. Few years later, a prospective study reported higher rate of preterm delivery in 87 euthyroid pregnant women with TPOAb as compared to pregnant women without TPOAb [2]. The postulated reasons, not mutually exclusive, for these associations appeared as follows: 1) unfavourable direct effect of thyroid antibodies at placental level; 2) subtle thyroid insufficiency; 3) thyroid autoimmunity as marker of an unfavourable autoimmune environment. Since then, a growing number of studies have tried to elucidate whether these associations were consistent and explored whether levothyroxine (LT4) treatment might be beneficial in reducing these pregnancy complications. Small randomised control trials and several meta-analyses confirmed that thyroid autoimmunity and subclinical hypothyroidism were strongly associated with miscarriage and preterm delivery and suggested that LT4 treatment may attenuate these risks [3–7]. A decisive answer in this controversial field recently came from a double-blind, placebo-controlled trial (the so called “TABLET” study), which aimed at investigating whether LT4 treatment would increase live-birth rates among euthyroid women with TPOAb and a history of miscarriage or infertility [8].

## Main text

The participants were recruited from 49 hospitals across the United Kingdom. As for the study protocol, women were eligible for enrolment if they were 16 to 40 years of age, had a history of miscarriage or infertility, and were trying to conceive in the subsequent 12 months (either naturally or through assisted conception). The screening stage involved blood tests for TPOAb, TSH (normal range 0.44 to 3.63 mIU/L) and FT4 (normal range 10.0–21.0 pmol/L). Participants were randomly assigned pre-conception in a 1:1 ratio to receive either 50 μg of LT4 or placebo once a day. After randomization, women underwent a 12-month evaluation and once pregnant, the women had three trial visits, which included thyroid-function testing, at 6–8 weeks, 16–18 weeks, and 28 weeks. A total of 19,556 women underwent screening tests, 1420 were eligible for enrolment, and the 952 were randomly assigned to receive LT4 treatment (476 women) or placebo (476 women). In patients with thyroid function test results outside of assay-specific reference limits, the trial agent was discontinued. 56.6% in the LT4 group and 58.3% in the placebo group became pregnant and the live birth rates were similar in the two groups (37.4% vs 37.9%, respectively). There was also no difference in preterm delivery rate and other maternal and neonatal outcomes between the two groups.

Thus, this UK study rejected the postulated benefits of treating euthyroid pregnant women with chronic autoimmune thyroiditis. The study confirmed the findings of another recent Chinese study which, however, recruited only women undergoing in vitro fertilization and embryo transfer [9]. In this latter study too, women were randomized to receive or not to LT4 but those who were treated with LT4 did not show any additional benefit in miscarriage or live birth rate.

The most interesting aspect of the study by Dhillon-Smith is the idea of administering LT4 pre-pregnancy, in women who wished to conceive. This characteristic of the study would also imply that if LT4 treatment was not effective before conception, it is even less likely to be effective if it is started during pregnancy. From this point of view, the study takes a special value when compared with other multicentre trials aimed at investigating the role of LT4 in reducing potential adverse events associated with impaired thyroid function in pregnancy. It is worth noting that both the studies by Lazarus and Casey, which tested the effect of LT4 in pregnant women with subclinical hypothyroidism and/or isolated hypothyroxinemia, failed to observe any positive effect on intelligence quotient of offspring, and maternal and neonatal outcomes, but both studies recruited patients beyond the first trimester [10, 11].

In my opinion, three points relating to the study by Dhillon-Smith deserve some discussion: 1) the authors claimed that notwithstanding 20% lower than expected live birth rate, the 95% confidence interval for the primary outcome ruled out a clinically meaningful benefit, and therefore a potential reduction in power did not affect their inferences. But in addition to this, nearly 10% of patients in both groups discontinued the trial because their thyroid function tests during the study showed results outside the reference range and in the LT4 group about 14% of patients (7–19% at different time points) showed a poor compliance (ingested pills < 75% than expected). No one knows to what extent the final results could have been globally impacted because of the remarkably lower than expected live birth rate, number of patients who dropped out and poor compliance. 2) If thyroid function test resulted outside the normal range, the trial agent was discontinued. Nearly 10% in both groups discontinued the trial agent because of abnormal results of thyroid function tests (presumably for hyperthyroidism in the LT4 group and for hypothyroidism in the placebo group). As a result, essentially the study showed no differences between two groups of antibody positive patients, one of which treated with LT4, but both having thyroid function tests within the normal range; as a consequence, it is worth highlighting that the negative results of the study are restricted to patients who maintain a normal thyroid function before and throughout pregnancy but should not be extended to those who have or develop thyroid insufficiency. 3) Whether thyroid antibodies per se increase pregnancy loss and preterm delivery remains an unanswered question, as an antibody negative group is missing: as from the protocol of the study, we still do not know whether thyroid antibody positive women experience increased rates of pregnancy loss and preterm delivery as compared to those with negative antibodies. For sure, from the study results we can infer that LT4 treatment has no positive influence on the general immune regulation, on the fetal-maternal interface (placenta), on trophoblast and decidual cell behaviour, and that it does not matter whether thyroid hormone levels are in the upper or lower limit of the reference range.

An issue that is still controversial and that may have had an influence on results of published trials in the field, is the different normal reference range that was adopted in each study [3, 4, 8–12]. This variability introduced substantial differences in the definition of hypothyroidism and consequently in the number of subjects submitted or not to treatment [13]. Differences may also be found in hypothyroxinemic women, sometimes considered together with those having elevated TSH, and sometimes evaluated as a separate category. Finally, remarkable differences regarding the administered dose of LT4 and the time of LT4 initiation, make results globally difficult to be compared and interpreted, still leaving margins of uncertainty.

At the moment, the study by Dhillon-Smith definitely supports a conservative approach when dealing with patients in childbearing age suffering from chronic autoimmune thyroiditis. Guidelines released by the American Thyroid Association in 2017 stated that there was insufficient evidence to conclusively determine whether LT4 therapy decreases pregnancy loss and pre-term delivery, but administration of LT4 may be considered [14]; but The TABLET study puts aside such a therapeutic option. However, as studies have demonstrated that women with thyroid autoimmunity are prone to develop hypothyroidism during pregnancy and that elevated TSH concentrations have been associated with pregnancy loss, it is reasonable to monitor these patients and treat them when TSH exceeds 4.0 mIU/L [2, 4, 15].

Another ongoing trial (T4-LIFE), is investigating the effect of LT4 administration on live birth rate in euthyroid TPOAb positive women with recurrent miscarriage. Also in this multicenter randomized, double blind placebo controlled trial, the authors will assess whether the treatment administered before pregnancy will improve the live birth rate. As the design of the T4-LIFE is very similar to the TABLET study, it will be interesting to see if the TABLET’s results will be confirmed [16].

Another implication from the TABLET study concerns the ongoing debate about screening women for thyroid dysfunction in early pregnancy. One of the criteria that needs to be met when implementing a screening strategy is that there should be agreed evidence-based policies covering which individuals should be offered treatment. In this light, results of the study by Dhillon-Smith clearly suggests a “wait and see” strategy for euthyroid TPOAb positive patients and states that LT4 should not be offered. Globally considering the fundamental trials published in the last few years, it is worth remembering the prophetic observations made by Laurberg et al. [17]. In a review published several years ago the authors observed that “the association found in several studies between small thyroid test abnormalities and pregnancy complications may be due to confounding, and thyroid hormone therapy will have no effect. Screening and therapy for overt thyroid dysfunction in early pregnancy may be indicated, rather than focusing on identifying and treating small aberrations in thyroid function tests.”

## Conclusion

The study shows that treating women suffering from chronic autoimmune thyroiditis with LT4, even before pregnancy, does not increase live birth rate. This finding supports a conservative approach when dealing with such patients in childbearing age or in early pregnancy.

## Data Availability

Not applicable.
